# Circular RNA Formation and Degradation Are Not Directed by Universal Pathways

**DOI:** 10.3390/ijms26020726

**Published:** 2025-01-16

**Authors:** Arvind Srinivasan, Emilia Mroczko-Młotek, Marzena Wojciechowska

**Affiliations:** Department of Rare Diseases, Institute of Bioorganic Chemistry, Polish Academy of Sciences, Noskowskiego 12/14, 61-704 Poznan, Poland; asrinivasan@ibch.poznan.pl (A.S.); emroczko@ibch.poznan.pl (E.M.-M.)

**Keywords:** circular RNAs, back-splicing, alternative splicing, *cis*-regulatory elements, *trans*-acting factors

## Abstract

Circular RNAs (circRNAs) are a class of unique transcripts characterized by a covalently closed loop structure, which differentiates them from conventional linear RNAs. The formation of circRNAs occurs co-transcriptionally and post-transcriptionally through a distinct type of splicing known as back-splicing, which involves the formation of a head-to-tail splice junction between a 5′ splice donor and an upstream 3′ splice acceptor. This process, along with exon skipping, intron retention, cryptic splice site utilization, and lariat-driven intron processing, results in the generation of three main types of circRNAs (exonic, intronic, and exonic–intronic) and their isoforms. The intricate biogenesis of circRNAs is regulated by the interplay of *cis*-regulatory elements and *trans*-acting factors, with intronic Alu repeats and RNA-binding proteins playing pivotal roles, at least in the formation of exonic circRNAs. Various hypotheses regarding pathways of circRNA turnover are forwarded, including endonucleolytic cleavage and exonuclease-mediated degradation; however, similarly to the inconclusive nature of circRNA biogenesis, the process of their degradation and the factors involved remain largely unclear. There is a knowledge gap regarding whether these processes are guided by universal pathways or whether each category of circRNAs requires special tools and particular mechanisms for their life cycles. Understanding these factors is pivotal for fully comprehending the biological significance of circRNAs. This review provides an overview of the various pathways involved in the biogenesis and degradation of different types of circRNAs and explores key factors that have beneficial or adverse effects on the formation and stability of these unique transcripts in higher eukaryotes.

## 1. Introduction

Circular RNAs are a distinct category of single-stranded transcripts that form covalently closed loop structures, ensuring longer half-lives compared to linear RNAs. CircRNAs were first discovered in 1976 as viroids [1], and their first detailed analysis was reported in the mid-1990s in mice [2]. Despite their early discovery, circRNAs were long considered rare events and classified as splicing errors [3]. However, the advent of high-throughput sequencing technologies over the past decade has led to the rediscovery of these transcripts across diverse species, including humans, animals, and plants, with growing insights into their biological significance [3,4,5,6,7,8].

CircRNAs are derived from protein-coding and non-coding regions of the genome and display considerable diversity in length and structure [9]. Based on their composition, circRNAs are classified into three major types, i.e., exonic (E-circRNAs), intronic (I-circRNAs), and exonic–intronic (E-I circRNAs) [10,11]. A particular type of splicing known as back-splicing is engaged in their generation, which involves the formation of a head-to-tail back-splice junction (BSJ) between a 5′ splice donor and an upstream 3′ splice acceptor. Several mechanisms have been proposed to explain the biogenesis of circRNAs, including the circularization of a single or many exons facilitated by nearby repetitive sequences in flanking introns, the use of exonic and intronic cryptic splice sites, inefficient debranching of intronic lariats, and intron retention (IR) in linear transcripts [12,13,14,15,16,17]. Nevertheless, the regulation of back-splicing is known to be influenced by both *cis*-regulatory elements and *trans*-acting RNA-binding proteins, which can modulate the efficiency of RNA circularization. The plethora of circRNAs is thought to be a result of either co-transcriptional or post-transcriptional mechanisms of their biogenesis. Earlier studies suggested that circRNAs are co-transcribed with their linear counterparts, as forward splicing and back-splicing may compete with each other during RNA processing [18]. Conversely, other research indicated that a stable 3′-end of pre-mRNA is necessary for circRNA formation, implying that this process may also occur post-transcriptionally [19]. Similarly to the inconclusive nature of circRNA biogenesis and the factors involved, the process of their degradation remains unclear regardless of multiple pathways and hypotheses being forwarded [20]. It remains to be determined whether the process is guided by a universal decay pathway or whether each category of circRNAs requires special tools and particular mechanisms for their degradation. CircRNAs’ structural diversity and higher stability than their linear counterparts predispose them to perform various cellular functions, playing either cis or trans roles when acting as transcription regulators or as competing endogenous RNAs to bind microRNAs (miRNAs) (RNA sponges) or RNA-binding proteins (protein sponges), and thus to modulate their local free concentration. Moreover, circRNAs might encode proteins with functions distinct from those of their canonical linear counterparts [21,22,23]. The biological significance of circRNAs has also been implicated in their roles as putative disease biomarkers, including neurological and cardiovascular illnesses and numerous types of cancer [10,24,25,26,27]. Nevertheless, their functions in eukaryotes still remain more or less elusive, although there has been a growing amount of data that circRNAs may fulfill important functions.

This review provides a comprehensive overview of various pathways involved in the biogenesis and degradation of different types of circRNAs and explores *cis*- and *trans*-acting factors’ beneficial or adverse effects on the formation and stability of these peculiar transcripts in higher eukaryotes.

## 2. Alternative Splicing and circRNA Isoforms

The discovery of circRNAs has greatly enriched the transcriptome by unveiling a diverse and widespread group of transcripts. These circRNA isoforms often arise from the alternative splicing of linear pre-mRNA, highlighting the complex interplay between alternative and back-splicing within genomic regions. A well-known example of this splicing interaction is the creation of single-exon circRNAs from linear transcripts that undergo exon-skipping events (cassette exons, CEs). The skipped exons are then circularized through an additional splicing mechanism. This phenomenon was first identified by Zaphiropoulos, who observed circularized exons in the rat cytochrome P450 2C24 [28].

Further research in humans and mice has revealed a similar pattern in many genes, suggesting that the circularization of cassette exons is a widespread and common process [29,30].

**Figure 1:**
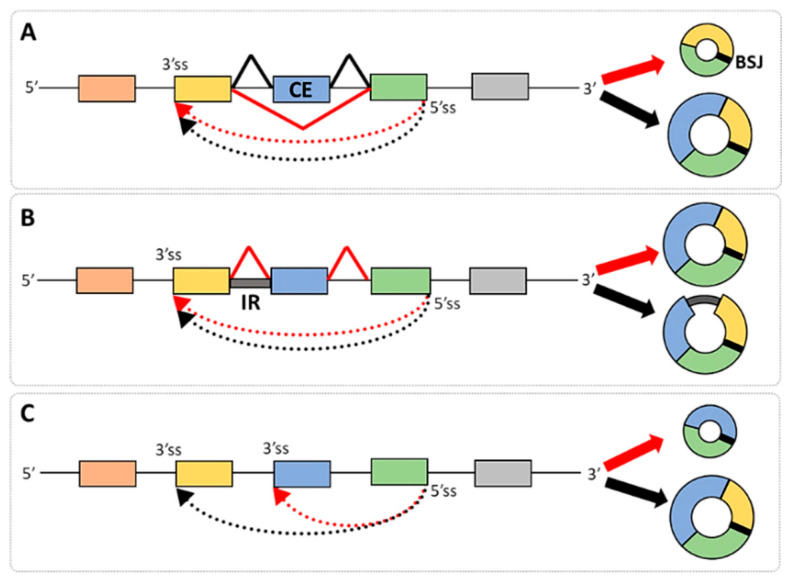
Formation of circRNA isoforms. (**A**,**B**) circRNA isoform formation is a result of the alternative splicing of host linear RNAs, which may include cassette exon events (CEs) or intron retention (IR). (**C**) circRNA isoforms may also originate from the alternative back-splice site selection, either 3′ or 5′. Filled rectangles indicate different exons; black lines indicate introns; black and red dashed arrows represents the process of forming a BSJ; black and red filled arrows indicate circRNA isoforms; BSJ is indicated by a thick black line in depicted circRNAs. Graphics prepared using Microsoft PowerPoint.

However, other types of alternative splicing give rise to diverse isoforms of linear transcripts from a single gene (i.e., mutually exclusive exons, alternative 5′ and 3′ splice sites, and intron retention could also contribute to the variety of circRNA isoforms) [31]. A characteristic feature of such isoforms is their identical back-splice junction but different sequence compositions [32,33] (Figure 1A,B). However, the formation of circRNA isoforms could also be achieved by the utilization of alternative BSJs during their biogenesis and may involve either the 5′ splice donors or the 3′ splice acceptors [32,33,34] (Figure 1C). Research in many laboratories has shown that over half of circRNA-producing loci can generate multiple isoforms through alternative splicing, with significant implications for many disorders [16,35]. While most human circRNAs originate from multi-exonic genes and exhibit diversity through alternative splicing, the full extent of the splicing in circRNAs relative to linear mRNAs is not fully explored yet. The growing understanding of circRNA isoform diversity suggests that these molecules may play a significant role in disease mechanisms. However, their full implications in pathology remain underexplored, highlighting a critical gap in current research that warrants further investigation. Recently, the CircSplice algorithm [36] has enabled the identification and comparison of circRNA splicing events, showing promise in characterizing disease- and tissue-specific circRNA alternative splicing and offering valuable insights into circRNA regulation.

## 3. Types of Circular Transcripts

**Figure 2:**
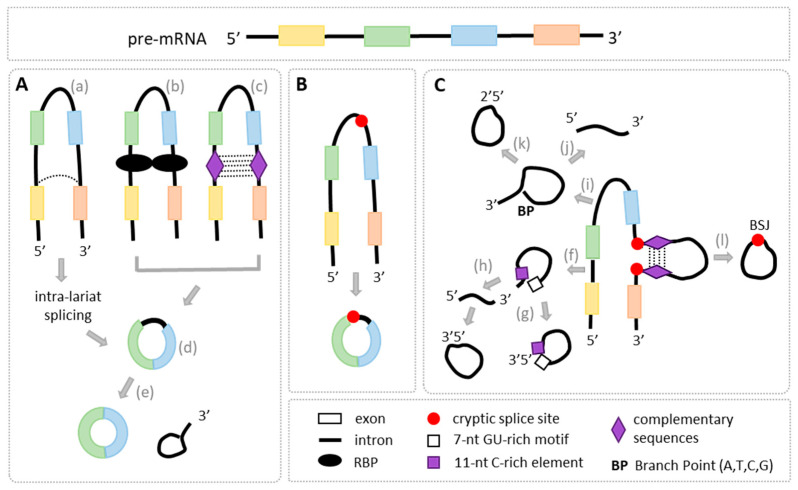
Biogenesis of different types of circRNAs. (**A**) Three models of E-circRNA biogenesis: (a) lariat-driven circularization linked with canonical exon skipping. (b) During this process, the intra-lariat is spliced via RNA-binding protein (RBP)-driven circularization and (c) via base-pairing-driven circularization of complementary sequences in flanking introns; as an outcome, an (d) E-I-circRNA-containing whole intron is synthesized or, (e) after the final intron removal, lariat and E-circRNA are formed as the final products. (**B**) Biogenesis of E-I circRNAs. The usage of cryptic donor and acceptor splice sites in introns results in their complete and/or partial retention in the final circRNA products. (**C**) Biogenesis of I-circRNAs. The formation of I-circRNAs depends on intron length. (f) Short introns (0.1–0.5 kb) containing a 7 nt GU-rich motif and an 11 nt C-rich element, (g) if they contain a cytosine (“C”) in the branch point (BP) instead of the canonical adenine, they avoid debranching and degradation. These I-circRNAs form a 3′-5′ phosphate link after nucleophilic attack on the 2′-5′ phosphodiester bond of the lariat. (h) Another method is a two-step process in which the lariat structure is debranched and subsequently re-ligated to form a circRNA. (i) Lariats created from long introns (>10 kb) (j) may either be linearized or degraded by DBR1 or, (k) following 3′-end trimming, they may become stable circRNAs. (l) I-circRNAs can also be formed through non-canonical splice sites within introns or the formation of secondary structures. Graphics prepared using Microsoft PowerPoint.

Based on the analysis of RNA-seq data deposited in various circRNA databases, there are three major categories of circular transcripts, i.e., E-circRNAs, I-circRNAs, and E-I circRNAs (Table S1). The most studied and best characterized are E-circRNAs composed of either single or multiple exons; they are the major group of circular transcripts found in different organisms, tissues, and cell types [13,37,38,39]. In most cases, their biogenesis involves the utilization of canonical splice donors and acceptors; however, it may also involve the interaction between cryptic intra-exonic and conventional splice sites, leading to the generation of circRNAs with a partial exon sequence [14]. Several models have been proposed to explain the origin of E-circRNAs. One of them, known as the exon-skipping model, suggests that circRNAs arise from an exon-containing lariat formed during the splicing of a pre-mRNA intermediate, encompassing a skipped exon (Figure 2A) [4]. Another model involves the re-splicing of mature mRNA when initial splicing removes canonical splice sites, and back-splicing occurs at cryptic splice sites [40]. Additionally, the circularization of exons can be facilitated by the pairing of flanking introns when their complementary sequences bring splice sites closer together [4,7,19]. The same effect is achieved via intronic binding of RBPs, which enhances the physical contact of downstream and upstream introns (Figure 2A). More detailed characteristics of factors that affect E-circRNA generation are described in the next chapter.

Studies on I-circRNAs have not been conducted as systematically as those on E-circRNAs, resulting in the identification of less than two hundred intron-derived transcripts to date when compared with thousands of exon-built circRNAs [41]. Based on these studies, two models for intron circularization have been proposed [42]. The first model involves a two-step process in which the lariat structure formed during splicing is debranched and subsequently re-ligated to form a circRNA (with a 3′-5′ covalent link) (Figure 2C). The second model results from a failure of a full debranching reaction leading to a nucleophilic attack on the 2′-5′ phosphodiester bond of the lariat, directly creating the 3′-5′ linkage. The first evidence of intronic circular transcripts was reported by Zhang and colleagues, who found that intron lariats containing an 11 nt C-rich element near the 5′ splice site and a 7 nt GU-rich region close to the branch point can produce I-circRNAs and escape debranching [43]. Several studies have demonstrated that circRNAs derived from lariats originate from introns that do not utilize an adenosine at the branch point, but rather a “C” and, less frequently, a “G” or “T” [44,45,46,47,48]. The lariat debranching enzyme (DBR1) is incapable of hydrolyzing the 2′-5′ bond in these residues, resulting in their accumulation and possibly directing them by yet-unknown mechanisms to utilization inside the cells. In addition to the above, I-circRNAs can be formed through intra-intronic cryptic splice sites [14]. When a cryptic donor splice site within an intron pairs with a cryptic acceptor splice site within the same or different intron, the resulting back-splicing leads to the formation of a circRNA that contains a partial intron sequence [14] (Figure 2C).

E-I circRNAs are a subclass of circRNAs characterized by the presence of one or more introns or their fragments (Figure 1B and Figure 2B). Their generation may rely on the utilization of cryptic splice sites in introns leading to a partial IR in the final RNA products (Figure 2B). Alternatively, as illustrated in Figure 1B, the entire intron may be retained in the circular RNA, resembling IR events in linear transcripts [15,17]. However, the global features of E-I circRNAs remain largely unexplored, and the regulation of the IR in these transcripts as well as their functionality require more systematic analysis. A recent study that used newly developed methods of finding E-I circRNA from the paired-end (FEICP) of high-throughput sequencing data revealed that intron-holding circRNAs compared to E-circRNAs are longer, nuclear-localized, tissue-specific, and enriched in the brain [49]. Moreover, introns retained in circRNAs are shorter than regular introns, have weaker splice site strength, and have higher GC content. Compared with retained introns in linear transcripts, circRNA IRs are more likely to form secondary structures and show greater sequence conservation, likely affecting their stability and modifying functionality.

## 4. Regulators of circRNA Biogenesis

The back-splicing mechanism of circRNA generation is governed by both *cis*-regulatory elements and *trans*-acting factors (Tables S2 and S3) [18,43,50,51,52]. This intricate process ensures the precise control of circRNA formation, contributing to their tissue-specific expression and functional diversity.

### 4.1. cis-Regulatory Elements

#### 4.1.1. The Features of Introns and Exons Participating in circRNA Generation

Regardless of the lack of a unified mechanism that accounts for transcript circularization, it appears that the collaboration between an exon and its flanking introns enables circRNA formation [2,4,19,24,53]. Capel and colleagues first proposed this idea in the 1990s, suggesting that inverted repeats in the introns flanking the Sry exon help facilitate circularization [2]. This hypothesis was later supported by the identification of over 15.5 kb of nearly complementary sequences flanking the Sry locus. Although such extensive repeats are not abundant throughout the genome, the authors highlighted the role of *cis*-elements in the process of RNA circularization. Further research based on large-scale genomic sequencing has revealed that in human introns surrounding circularized exons, there are numerous inverted Alu repeats of approximately 300 nucleotides [54,55]. Initially, it was believed that multiple repeats are necessary for circRNA formation [4]. However, research conducted by Zhang and colleagues, who modified sequences of introns flanking the BSJ of POLR2A circRNA, found that differential pairing of inverted Alu repeats was responsible for a variety of circularized transcripts from a single gene [53]. Similarly, Liang and colleagues found that short inverted repeats (30–40 nucleotides) and miniature introns (<100 nucleotides) were sufficient for the circularization of exons in circRNAs originating from ZKSCAN1, HIPK3, and EPHB4 [19]. Ottesen and colleagues further explored this concept by identifying several circRNAs from the human survival motor neuron (SMN) gene, which contains numerous Alu elements spanning a significant portion of the transcribed region. Importantly, these authors observed that fewer circRNAs from Smn in mice when compared to the human gene were attributed to the lower proportion of Alu elements [56]. A comparative study by Tang and colleagues revealed that only a small fraction of human circRNAs were conserved in mice, with the majority of circRNAs in both species exhibiting species-specific expression patterns, with a higher percentage observed in humans. Overall, this leads to the conclusion that the accumulation of Alu repeats in humans has played a significant role in enhancing the diversity of circRNAs throughout evolutionary history [57].

However, regardless of the features described above, not all exons undergo circularization. As reported, only about 40% of circRNAs detected in human fibroblast cells are expressed from loci containing Alu repeats in their flanking introns, and merely 20% of them are complementary [4]. The absence of inverted repeats in many introns flanking circularizable exons suggests the presence of additional mechanisms of exon circularization. With the exception of a gene’s first and last exons, every other exon in the genome has splicing signals at its 5′ and 3′ ends and, theoretically, can circularize. However, the terminal exons are often being utilized through cryptic splice sites. Also, the effectiveness of exon circularization has been linked with their size, and this subject remains a matter of debate since some data have suggested more efficient circularization of longer exons, while other data have proposed the opposite, claiming that circles form more readily from smaller exons [53,58]. Importantly, many exons forming circRNAs are not among alternatively spliced cassette exons, suggesting that their peculiar genomic features, along with the availability of splicing factors, play a role when it comes to making decisions about forward or back-splicing [59]. Therefore, the choice between the production of a linear mRNA and a circRNA must be tightly regulated, as it fundamentally changes the functional output of a gene. Nevertheless, detailed mechanistic models of how circularization generally occurs are still missing, limiting our understanding of how these transcripts are regulated and how they function.

#### 4.1.2. The Features of Canonical and Cryptic Splice Sites Involved in BSJ Formation

Most circRNAs are generated at canonical splice sites through the U2 spliceosome machinery [37,60]. Studies have shown that mutating the splice sites involved in both forward and back-splicing reduces the production of both linear and circular transcripts from the same gene locus, emphasizing the need for specific splice signals in the circularization process [18,61,62]. Nevertheless, non-canonical donor and acceptor splice sites are also found in BSJs, as shown in circRNAs originating from internal exons and introns in humans and mice [63]. Another feature affecting variations in splicing efficiency and the formation of circRNAs is the strength of splice sites (Table S2). Zhang and colleagues identified exons forming circRNAs with splice site strengths comparable to constitutive exons and those subjected to exclusion [33]. Di Liddo and colleagues reported that the 5′ splice sites of introns at donor back-splice sites exhibited significantly higher strength than 5′ splice sites at flanking or randomly selected sites not involved in back-splicing [64]. Similar findings were observed in hnRNPM-originating back-spliced exons, which had stronger 5′ splice sites compared to their corresponding linear exons.

Most exonic circRNAs are composed of one or more exons [13]. Typically, only internal exons of a gene undergo back-splicing, as the first and last exons are excluded due to the absence of, respectively, acceptor and donor splice sites [53,65]. However, deviations from this rule have been reported. For instance, Hu and colleagues observed that in the UDP glucuronosyltransferase 1 family, polypeptide A cluster (UGT1A), the first 317 nucleotides of the last exon were involved in back-splicing to form circRNA through a cryptic exonic donor splice site. Similarly, a fragment of the last exon of UDP Glycosyltransferase 8 (UGT8) was circularized via a cryptic donor splice site [14]. These non-canonical splice sites in the last exons exhibited the conserved “gt” dinucleotides, adhering to the canonical splicing rule. Other studies have also identified cryptic donor and acceptor splice sites within exons, which are frequently utilized in back-splicing to generate E-circRNAs containing partial exon sequences (Figure 2, Table S2). Non-canonical splice sites within introns are characteristic of circRNAs formed entirely from intronic sequences or those of mixed composition, i.e., exonic–intronic [49]. In these cases, back-splicing occurs when a cryptic donor splice site within an intron pairs with a cryptic acceptor splice site within the same or a different intron, giving rise to a circRNA composed exclusively of intronic regions [14]. Similarly, the formation of E-I circRNAs involves cryptic splice sites within exons or introns that are not typically used in canonical splicing (Figure 2). The formation of these circRNAs highlights the complexity of back-splice junction (BSJ) formation, emphasizing the interaction between canonical and cryptic splice sites, with variations in splice site potency contributing to circRNA diversity. This intricate regulation of RNA splicing plays a crucial role in the biogenesis and function of circRNAs.

### 4.2. trans-Regulatory Factors

Back-splicing is a complex process regulated by numerous *trans*-acting factors, including RBPs, which can either promote or inhibit circRNA formation (Table S3). The first protein identified as a facilitator of exon circularization was the splicing factor muscleblind (Mbl) in Drosophila and the vertebrate homolog muscleblind-like protein 1 (MBNL1) [18]. Later works in different systems and organisms have identified other RBPs regulating RNA circularization, including adenosine deaminases acting on RNA (ADAR), quaking (QKI), FUS, ATP-dependent RNA helicase A (DHX9), and SR-rich proteins [7,50,51,66]. The splicing factor Mbl/MBNL1 was shown to mediate one of the mechanisms of RNA circularization via binding in highly conserved intronic sites and promoting circularization of its own second exon [18]. Introns flanking this exon of Mbl contain short inverted sequences that are likely to stabilize inter-intron interactions, which are too weak to promote exon circularization in the absence of its binding [18]. This suggests that Mbl binding to the flanking introns facilitates intron–intron interactions and promotes circularization. In Drosophila, the protein strongly binds to circMbl, indicating the existence of a regulatory loop between these two molecules. Additionally, as experimentally shown, the Mbl molecules may dimerize, facilitating circRNA formation by bringing the two ends of the exon together (Figure 2A) [67]. Other RBPs, such as QKI, promote global circRNA production by binding to intronic sequences surrounding the circularized exons, and, similarly to Mbl, they might dimerize and facilitate RNA circularization [66,68]. The mechanism of action of Splicing Factor Proline and Glutamine Rich (SFPQ) relies on interaction with long flanking introns containing distal inverted Alu repeat elements [22]. In addition, FUS regulates circRNA formation in neurons, and its mutation may contribute to the pathogenesis of amyotrophic lateral sclerosis (ALS) [69]. Moreover, the biosynthesis of some E-circRNAs was shown to be regulated by a combination of different RBPs, such as hnRNPs and SR proteins, suggesting that the circularization efficiency can result from the integration of several signals [51]. The binding of RBPs and their dimerization, along with the presence of long inverted repeats in introns, promote intron–intron interactions that facilitate circRNA formation. Also, particular physiological circumstances that involve the formation of dsRNA structures may alter circRNA biogenesis. In fact, RNA editing by the double-stranded RNA-specific ADAR modulates circRNA biogenesis in human and mouse cell cultures and in fruit flies [7,70]. The ADAR can inhibit circularization by disrupting inverted-repeat Alu elements through adenosine-to-inosine editing, which destabilizes RNA pairs [7]. In addition, DHX9, which interacts with an interferon-inducible isoform of ADAR (p150) and disrupts RNA secondary structures like those based on Alu inverted repeats, also suppresses circRNA production.

While most RNA circularization studies have focused on the regulation of back-splicing in the generation of E-circRNAs, little is known about the modulation of the production of E-I circRNAs and the mechanism of IR in these transcripts. The most recent study by Zhong and colleagues suggested that while there is an array of RBPs involved in the regulation of the expression of E-I circRNAs, the majority of these proteins play suppressive roles in the circularization process [49]. One of the RBPs identified by these authors was serine and arginine-rich splicing factor 1 (SRSF1) inhibiting a significant fraction of E-I circRNAs by binding to both the IR and circularized exons. Interestingly, previous studies showed that SRSF1 was also involved in the regulation of IR in linear transcripts; however, on comparing these results of IR events, the authors found a limited overlap for either introns or parental genes between regulated circular and linear RNAs [49,71,72]. This suggests that distinct regulatory mechanisms operate on circular and linear IR events. Further investigations are needed to understand the roles of SRSF1 and its cofactors in modulating the generation of E-I circRNAs.

## 5. Degradation of Circular RNAs

**Figure 3:**
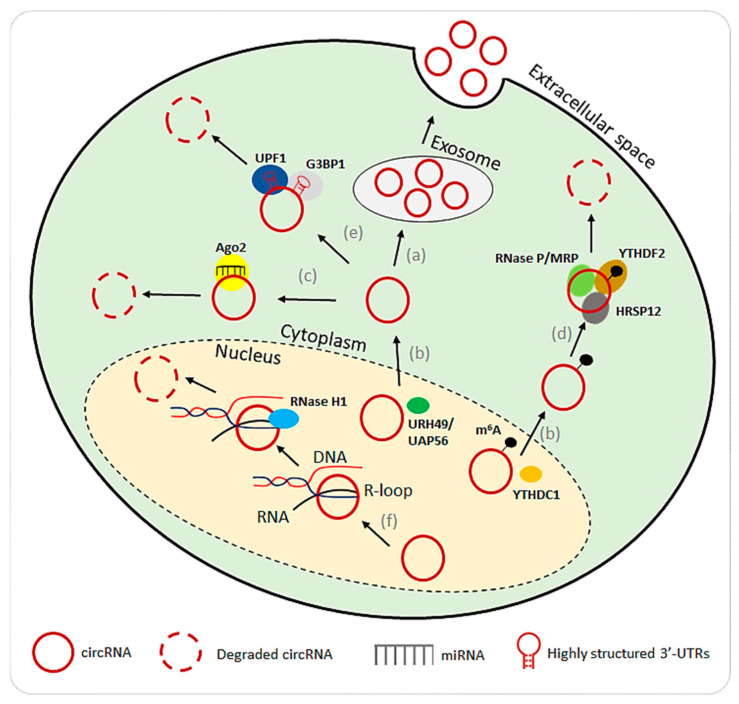
A model depicting different mechanisms of circRNA degradation. (a) elimination of circRNAs by means of extracellular vesicles, such as exosomes; (b) nucleocytoplasmic transport of circRNAs mediated by URH49/UAP56 and m6A modification by YTHDC1; (c) Ago2-mediated degradation in the presence of a circRNA-miRNA complex; (d) m6A-mediated regulation of degradation through the YTHDF2-HRSP12-RNase-P/MRP interaction; (e) degradation triggered by RNA structure via the recruitment of UPF1 and G3BP1; (f) RNase H1-driven degradation through the formation of R-loops of RNA-DNA hybrids. Graphics prepared using Microsoft PowerPoint.

CircRNA degradation is a complex process that is not yet well understood. One of the mechanisms involves the export of circRNAs from cells through extracellular vesicles, particularly exosomes. By exporting circRNAs, cells regulate their intracellular concentrations, which may suggest a mechanism of circRNA clearance (Figure 3a) [73]. This process of extracellular transport also raises the possibility that circRNAs contribute to intercellular communication since exosomes carry them between cells. Another critical factor in circRNA stability and degradation is nuclear export. Proteins such as helicase at 25E (Hel25E), YTH domain-containing protein 1 (YTHDC1), and ATP-dependent helicases (e.g., URH49, UAP56) are involved in the nuclear-to-cytoplasmic transport of circRNAs (Figure 3b) [74,75]. Once transported to the cytoplasm, circRNAs engage in cytoplasmic processes but also become susceptible to degradation, making nuclear export a crucial step in their life cycle and stability. Yet another pathway of circRNA degradation involves the Argonaute 2 (AGO2) protein, which mediates the cleavage of circRNAs through miRNA interactions (Figure 3c). For instance, miR-671 and miR-1224 guide Ago2 to degrade specific circRNAs, such as circRNA-CDR1as and circRNA-Filip1 [76,77]. This reveals a connection between circRNA turnover and the broader miRNA regulatory network, highlighting that circRNAs not only sponge miRNAs but may also be targeted for degradation through these interactions.

RNA modification via N6-methyladenosine (m6A) adds another layer of circRNA regulation. It was experimentally shown that some circRNAs, such as circRNA-SORE and circ3823, modified with m6A, are targeted for degradation through the YTHDF2-HRSP12-RNase P/MRP pathway (Figure 3d) [78,79]. The YTHDF2 recognizes m6A-marked circRNAs and recruits HRSP12, which facilitates their degradation by RNase P/MRP [80,81]. This pathway shows how RNA modifications influence circRNA stability and decay in response to specific cellular signals. CircRNAs could also be degraded by structure-mediated RNA decay (SRD). RNA-binding proteins, including Up-frameshift suppressor 1 homolog (UPF1) and Ras-GapSH3 domain-binding protein 1 (G3BP1), bind to highly structured circularized regions and recruit decay machinery to facilitate degradation (Figure 3e) [82]. This pathway underscores the importance of circRNA secondary structures in determining their stability and susceptibility to degradation, thus requiring knowledge of their full composition. Lastly, degradation via RNase H1 was indicated in targeting circRNAs (Figure 3f) [83]. This process relies on the formation of R-loops of RNA-DNA hybrids, which are degraded by the endonuclease.

## 6. Conclusions and Future Directions

The studies described in this review indicate that the splicing-related mechanisms of circRNA biogenesis, which involve the formation of a head-to-tail splice junction between a 5′ splice donor and an upstream 3′ splice acceptor, are the best characterized in exon circularization. However, the intronic and exonic–intronic classes of circRNAs have not been systematically studied, and current knowledge about their genesis, function, and decay remains fragmentary. The role of cryptic splice sites in BSJ formation is also not fully understood, and their variability across tissues is unclear. Most studies in this area rely on in silico analysis rather than experimental validation. Therefore, more research is needed to elucidate the full nature of these transcripts, their diverse isoforms, and their roles in diseases associated with circRNA dysregulation. Recent advances in RNA sequencing technologies are expected to address these challenges by enabling a more comprehensive determination of their complete structure and sequence composition. Consequently, they should allow for a better understanding of circRNA cellular functions and help to decipher their degradation process, which remain highly elusive. This knowledge will serve as a gateway to a more complete understanding of circRNAs’ roles in different cells, tissues, and organisms.

## Supplementary Materials

The supporting information can be downloaded at https://www.mdpi.com/article/10.3390/ijms26020726/s1.
