# Supplementary material for: Circular RNA Formation and Degradation Are Not Directed by Universal Pathways
Source: Int J Mol Sci. 2025 Jan 16;26(2):726. doi: 10.3390/ijms26020726 (PMC11766002; doi:10.3390/ijms26020726)
Supplement: Supplementary file 1 [file ijms-26-00726-s001.zip › ijms-3387224-supplementary.pdf]

SUPPLEMENTARY TABLES

(Including a list of references)

Table S1: *Summary Features of CircRNA Types.*

CircRNA Type	Model of Biogenesis	Splice Site	CircRNA Composition	Intron Features	CircRNA Cellular Localization	CircRNA Degradation
Exonic	- exon-skipping<br>- re-splicing of mRNA<br>- intronic complementary sequences<br>- intronic binding of RBPs	- canonical<br>- cryptic	- single or multiple exons<br>- entire or partial exon sequence	- the presence of complementary sequences<br>- RBPs binding sites	cytoplasmic	- via RNase H<br>- small RNA-mediated degradation<br>- endoribonuclease RNase L [1]
Intronic	- debranching of the lariat and its subsequent re-ligation;<br>- failure of full debranching and nucleophilic attack on the 2'-5' phosphodiester bond of the lariat	- canonical<br>- cryptic	- lariat sequence without a tail<br>- partial intron sequence	- a lariat from an ~0.1–0.5 kb long intron with an unusual cytosine branch point<br>- A medium-sized intron of ~1–10 kb may be circularized via the canonical branch point<br>- large introns over 10 kb may have A, C, G or T in the BP	- nuclear<br>- cytoplasmic	- RNase H1 cleaves circRNAs that form DNA:RNA hybrids at their expression loci [2]
Exonic-Intronic	- utilization of cryptic splice sites in intron<br>- intronic complementary sequences<br>- intronic binding of RBPs	- canonical<br>- cryptic	- one or more exons; whole or partial sequences<br>- one or more retained introns; fragments or entire sequences	- short introns<br>- weak splice site strength<br>- high GC content	nuclear	- Combined features of other types results in mixed degradation pathway<br>- intron degradation by spliceosomal factors [3]

Table S2: *Cis*-Acting Elements Involved in CircRNA Biogenesis.

CIS-ACTING ELEMENT	SCHEMATIC REPRESENTATION	REFERENCE
Intron and Exon Lengths<br><br>Long flanking introns facilitate exons circularization; larger exons (>300 bp) are more susceptible to circularization	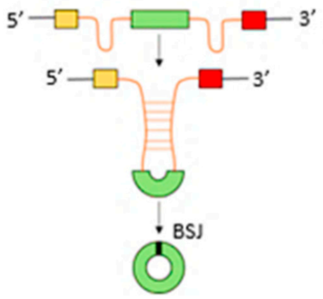	[4, 5, 6, 7, 8, 9]
Intronic Repetitive Sequences<br><br>The presence of Alu elements within flanking introns and their complementarity	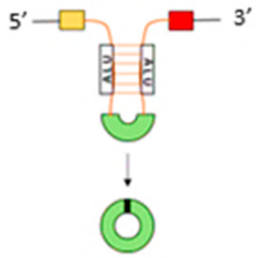	[7, 10, 11]
Dinucleotides at Splice Sites<br><br>Mostly canonical ag/gt splice signals; in rare cases non-canonical	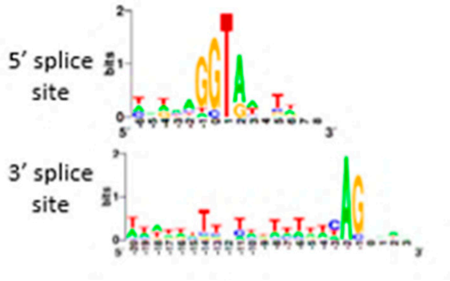	[12, 13, 14]
5′ and 3′ Splice Sites Strength<br><br>Back spliced exons with stronger 5' splice sites compared to their corresponding linear exons are prone to RNA circularization	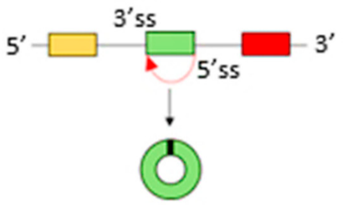	[15, 16]
Cryptic Splice Sites<br><br>The presence of cryptic splice sites within introns and exons are utilized	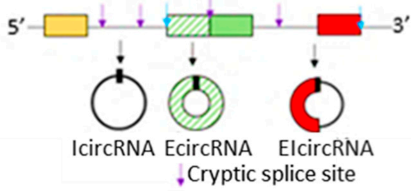	[17, 18, 19 ,20]

Table S3: *Trans*-Acting Proteins Involved In CircRNA Biogenesis, Inhibition and Degradation.

PROTEIN	TARGETED circRNA	FUNCTION*	REFERENCE
EIF4A3	cSERPINE2	F	[21]
	circ_0087429	I	[22]
	circLMNB2	F	[23]
	circTOLLIP	F	[24]
	circHomer1	F	[25]
	hsa_circ_0088088	F	[26]
	circSEPT9	F	[27]
	circEHMT1	F	[28]
	hsa_circ_0090081	F	[29]
	circEIF4A3	F	[30]
	hsa_circ_0049396	F	[31]
	circ_0059914	F	[32]
ESRP1	circDOCK1	F	[33]
	circ-TNPO3	F	[34]
	circZNF652	F	[35]
	circUHRF1	F	[36]
	circCAMSAP1	F	[37]
	circANKS1B	F	[38]
	circBIRC6	F	[39]
	circ_0068162	F	[40]
	circDOCK1	F	[41]
FUS	circCNOT6L	F	[42]
	circHIF1A	F	[43]
	circFGFR1	F	[44]
	circRHOBTB3	F	[45]
	Significantly down-regulated circRNAs upon FUS knockdown	F and I	[46]
QKI	circSlc17a5	F	[47]
	Specific circRNAs derived from Titin and Striatin	F	[48]
	circRNAs: POLE2, SMARCA5, OXNAD1, SHPRH, SMAD2, ATXN2, DOCK1, GNB1	F	[49]
PTBP1	circATIC	F	[50]
	circTOP1	F	[51]
HNRNPL	circANKRD42	F	[52]
	significantly up-and down-regulated circRNAs upon HNRNPL knockdown	F and I	[53]
QKI-5	hsa_circ_0098181	F	[54]
	circARFGEF2	F	[55]
ADAR1	circNEIL3	I	[56]
	circARSP91	I	[57]
USF1	circPRDM4	F	[58]
E2F6	hsa_circ_001726	F	[59]
FOXA1	circODC1	F	[60]

HUR	circ_0082319	F	[61]
ESRP1	circPTPN12	F	[62]
DNMT1	hsa_circ_401351	I	[63]
ZC3H13	hsa_circ_0101050	F	[64]
XBP1	circUbqln1	F	[65]
HNRNPD	circCDK1	I	[66]
DDX5	circPHF14	F	[35]
SRRM4	> 2000 circRNAs comprise canonical exonic and intronic sequences including circEIF4G3	F	[67]
DHX9	circRNAs containing inverted *Alu* repeats	I	[68]
NOVA2	circEfnb2	F	[69]
KAT5	circSMARCA5	F	[70]
E2F1	circSEPT9	F	[27]
SRSF10	circ-ATXN1	F	[14]
TNRC6A	circ0006916	F	[71]
RBM20	circRNAs originating from the I-band of the *Titin*	F	[72]
RBM3	SCD (stearoyl-CoA desaturase)-circRNA 2	F	[73]
NF90/NF110	circPOLR2A, circDHX34, circPDE3B	F	[74]
MBL/MBNL1	circMbl	F	[46]
c-Myb	circHIPK3	F	[75]
hnRNP and SR proteins	Laccase2 circRNA	F	[5]
RNA Pol II and U1 snRNP	circEIF3J, circPAIP2	F	[18]
RNA Pol II	ci-ankrd52,<br>ci-sirt-7	F	[76]
RNase L	Global circRNAs	D	[77]
Rrp44	Global circRNAs	D	[78]
GW182	circRNAs: CaMKI, Dbp80, uex, laccase2, Haspin, ps, pan, dati, nipped-B, CG42663, PlexA, Ect4, mnb	D	[79]
PKR	circPOLR2A	D	[77]
Ago2	CDR1as/ciRS-7	D	[80]
SFPQ	Distal-Alu-Long-Intron (DALI) circRNAs	F	[81]

*****, F-facilitate circRNA biogenesis; I-impede circRNA biogenesis; D-degrade circRNA

REFERENCES (to supplementary Tables S1, S2 and S3)

1. Patop, I. L., Wüst, S., & Kadener, S. Past, present, and future of circRNAs. The EMBO journal, 2019, 38(16):e100836.
2. Li, X., Zhang, J. L., Lei, Y. N., Liu, X. Q., Xue, W., Zhang, Y., Nan, F., Gao, X., Zhang, J., Wei, J., Yang, L., & Chen, L. L. Linking circular intronic RNA degradation and function in transcription by RNase H1. Science China. Life sciences, 2021, 64(11), 1795–1809.
3. Guria, A., Sharma, P., Natesan, S., & Pandi, G. Circular RNAs-The Road Less Traveled. Frontiers in molecular biosciences, 2020, 6, 146.
4. Chen, I., Chen, C.Y., Chuang, T.J. Biogenesis, identification, and function of exonic circular RNAs. Wiley Interdiscip Rev RNA, 2015, 6(5):563-79.
5. Kramer, M. C., Liang, D., Tatomer, D. C., Gold, B., March, Z. M., Cherry, S., & Wilusz, J. E. Combinatorial control of Drosophila circular RNA expression by intronic repeats, hnRNPs, and SR proteins. Genes & development, 2015, 29(20), 2168–2182.
6. Stagsted, L.V., Nielsen, K.M., Daugaard, I., Hansen, T.B. Noncoding AUG circRNAs constitute an abundant and conserved subclass of circles. Life Sci Alliance, 2019, 2(3):e201900398
7. Capel, B., Swain, A., Nicolis, S., Hacker, A., Walter, M., Koopman, P., Goodfellow, P., Lovell-Badge, R. Circular transcripts of the testis-determining gene Sry in adult mouse testis. Cell, 1993, 73(5):1019-30.
8. Ragan, C., Goodall, G.J., Shirokikh, N,E., Preiss, T. Insights into the biogenesis and potential functions of exonic circular RNA. Sci Rep, 2019, 9(1):2048.
9. Santer, L., Bär, C., Thum, T. Circular RNAs: A Novel Class of Functional RNA Molecules with a Therapeutic Perspective. Mol Ther, 2019, 27(8):1350-1363.
10. Jeck, W.R., Sorrentino, J.A., Wang, K., Slevin, M.K., Burd, C.E., Liu, J., Marzluff, W.F., Sharpless, N.E. Circular RNAs are abundant, conserved, and associated with ALU repeats. RNA, 2013, 19(2):141-57.
11. Liang, D., Wilusz, J.E. Short intronic repeat sequences facilitate circular RNA production. Genes Dev, 2014, 28(20):2233-47.
12. Starke, S., Jost, I., Rossbach, O., Schneider, T., Schreiner, S., Hung, L. H., & Bindereif, A. Exon circularization requires canonical splice signals. Cell reports, 2015, 10(1), 103–111.
13. Liang, G., Yang, Y., Niu, G., Tang, Z., Li, K. Genome-wide profiling of Sus scrofa circular RNAs across nine organs and three developmental stages. DNA Res, 2017, 24(5):523-535
14. Liu, X., Hu, Z., Zhou, J., Tian, C., Tian, G., He, M., Gao, L., Chen, L., Li, T., Peng, H., & Zhang, W. Interior circular RNA. RNA biology, 2020, 17(1), 87–97.
15. Di Liddo, A., de Oliveira Freitas Machado, C., Fischer, S., Ebersberger, S., Heumüller, A. W., Weigand, J. E., Müller-McNicoll, M., & Zarnack, K. A combined computational pipeline to detect circular RNAs in human cancer cells under hypoxic stress. Journal of molecular cell biology, 2019, 11(10), 829–844.
16. Ho, J. S., Di Tullio, F., Schwarz, M., Low, D., Incarnato, D., Gay, F., Tabaglio, T., Zhang, J., Wollmann, H., Chen, L., An, O., Chan, T. H. M., Hall Hickman, A., Zheng, S., Roudko, V., Chen, S., Karz, A., Ahmed, M., He, H. H., Greenbaum, B. D., … Guccione, E. HNRNPM controls circRNA biogenesis and splicing fidelity to sustain cancer cell fitness. eLife, 2021, 10, e59654.
17. Hu, D. G., Mackenzie, P. I., Hulin, J. A., McKinnon, R. A., & Meech, R. Circular RNAs of UDP-Glycosyltransferase (UGT) Genes Expand the Complexity and Diversity of the UGT Transcriptome. Molecular pharmacology, 2021, 99(6), 488–503.
18. Li, Z., Huang, C., Bao, C. et al. Exon-intron circular RNAs regulate transcription in the nucleus. Nat Struct Mol Biol, 2015, 22, 256–264.
19. Sunagawa, Y., Yamada, S., Sonohara, F. et al. Genome-wide identification and characterization of circular RNA in resected hepatocellular carcinoma and background liver tissue. Sci Rep, 2021, 11, 6016.
20. Rahimi, K., Færch Nielsen, A., Venø, M.T., Kjems, J.. Nanopore long-read sequencing of circRNAs. Methods, 2021, 196:23-29.

21. Zhou, B., Mo, Z., Lai, G., Chen, X., Li, R., Wu, R., Zhu, J., & Zheng, F. Targeting tumor exosomal circular RNA cSERPINE2 suppresses breast cancer progression by modulating MALT1-NF-κB-IL-6 axis of tumor-associated macrophages. Journal of experimental & clinical cancer research, 2023, 42(1), 48.
22. Yang, M., Hu, H., Wu, S., Ding, J., Yin, B., Huang, B., Li, F., Guo, X., & Han, L. EIF4A3-regulated circ_0087429 can reverse EMT and inhibit the progression of cervical cancer via miR-5003-3p-dependent upregulation of OGN expression. Journal of experimental & clinical cancer research, 2022, 41(1), 165.
23. Qiu, M., Chen, M., Lan, Z., Liu, B., Xie, J., Li, X. Plasmacytoma variant translocation 1 stabilized by EIF4A3 promoted malignant biological behaviors of lung adenocarcinoma by generating circular RNA LMNB2. Bioengineered, 2022, 13(4):10123-10140.
24. Liu, Y., Song, J., Zhang, H., Liao, Z., Liu, F., Su, C., Wang, W., Han, M., Zhang, L., Zhu, H., Zhang, Z., Liang, H., Zhang, L., Zhang, B., & Chen, X. EIF4A3-induced circTOLLIP promotes the progression of hepatocellular carcinoma via the miR-516a-5p/PBX3/EMT pathway. Journal of experimental & clinical cancer research, 2022, 41(1), 164.
25. Hafez, A. K., Zimmerman, A. J., Papageorgiou, G., Chandrasekaran, J., Amoah, S. K., Lin, R., Lozano, E., Pierotti, C., Dell'Orco, M., Hartley, B. J., Alural, B., Lalonde, J., Esposito, J. M., Berretta, S., Squassina, A., Chillotti, C., Voloudakis, G., Shao, Z., Fullard, J. F., Brennand, K. J., … Mellios, N. A bidirectional competitive interaction between circHomer1 and Homer1b within the orbitofrontal cortex regulates reversal learning. Cell reports, 2022, 38(3), 110282.
26. Liu, Q., Dong, H. EIF4A3-mediated hsa_circ_0088088 promotes the carcinogenesis of breast cancer by sponging miR-135-5p. J Biochem Mol Toxicol, 2021, 35(11):e22909.
27. Zheng, X., Huang, M., Xing, L., Yang, R., Wang, X., Jiang, R., Zhang, L., & Chen, J. The circRNA circSEPT9 mediated by E2F1 and EIF4A3 facilitates the carcinogenesis and development of triple-negative breast cancer. Molecular cancer, 2020, 19(1), 73.
28. Wang, Y., Zhang, Y., Qu, Y., Li, S., Xi, W., Liu, B., Ye, L. eIF4A3-mediated circEHMT1 regulation in retinal microvascular endothelial dysfunction in diabetic retinopathy. Microvasc Res, 2024, 151:104612.
29. Mou, Y., Lv, K. Extracellular vesicle-delivered hsa_circ_0090081 regulated by EIF4A3 enhances gastric cancer tumorigenesis. Cell Div, 2024, 19(1):19.
30. Li, Q., Wang, Z., Wang, J., Wang, J., Zheng, X., Li, D., Wang, Z., Li, J., & Li, Y. Regulatory feedback loop between circ-EIF4A3 and EIF4A3 Enhances autophagy and growth in colorectal cancer cells. Translational oncology, 2024, 46, 101996.
31. Zhou, Q., Cai, B., Liu, K., Chen, H. EIF4A3-Induced Upregulation of hsa_circ_0049396 Attenuates the Tumorigenesis of Nasopharyngeal Carcinoma by Regulating the Hippo-YAP Pathway. DNA Cell Biol, 2024, 10.1089/dna.2024.0119.
32. Yu, W., Chen, D., Ma, L., Lin, Y., Zheng, J., & Li, X. EIF4A3-Induced Circ_0059914 Promoted Angiogenesis and EMT of Glioma via the miR-1249/VEGFA Pathway. Molecular neurobiology, 2024, 10.1007/s12035-024-04319-w.
33. Liu, D., Dredge, B. K., Bert, A. G., Pillman, K. A., Toubia, J., Guo, W., Dyakov, B. J. A., Migault, M. M., Conn, V. M., Conn, S. J., Gregory, P. A., Gingras, A. C., Patel, D., Wu, B., & Goodall, G. J. ESRP1 controls biogenesis and function of a large abundant multiexon circRNA. Nucleic acids research, 2024, 52(3), 1387–1403.
34. Pan, X., Huang, B., Ma, Q., Ren, J., Liu, Y., Wang, C., Zhang, D., Fu, J., Ran, L., Yu, T., Li, H., Wang, X., Yang, F., Liang, C., Zhang, Y., Wang, S., Ren, J., Li, W., Wang, Y., & Xiao, B. Circular RNA circ-TNPO3 inhibits clear cell renal cell carcinoma metastasis by binding to IGF2BP2 and destabilizing SERPINH1 mRNA. Clinical and translational medicine, 2022, 12(7), e994.
35. Wang, X., Xu, C., Cai, Y., Zou, X., Chao, Y., Yan, Z., Zou, C., Wu, X., & Tang, L. CircZNF652 promotes the goblet cell metaplasia by targeting the miR-452-5p/JAK2 signaling pathway in allergic airway epithelia. The Journal of allergy and clinical immunology, 2022, 150(1), 192–203.

36. Wang, Y., Yan, Q., Mo, Y., Liu, Y., Wang, Y., Zhang, S., Guo, C., Wang, F., Li, G., Zeng, Z., & Xiong, W. Splicing factor derived circular RNA circCAMSAP1 accelerates nasopharyngeal carcinoma tumorigenesis via a SERPINH1/c-Myc positive feedback loop. Molecular cancer, 2022, 21(1), 62.
37. Zhou, C., Liu, H. S., Wang, F. W., Hu, T., Liang, Z. X., Lan, N., He, X. W., Zheng, X. B., Wu, X. J., Xie, D., Wu, X. R., & Lan, P. circCAMSAP1 Promotes Tumor Growth in Colorectal Cancer via the miR-328-5p/E2F1 Axis. Molecular therapy : the journal of the American Society of Gene Therapy, 2020, 28(3), 914–928.
38. Zeng, K., He, B., Yang, B. B., Xu, T., Chen, X., Xu, M., Liu, X., Sun, H., Pan, Y., & Wang, S. The pro-metastasis effect of circANKS1B in breast cancer. Molecular cancer, 2018, 17(1), 160.
39. Yu, C. Y., Li, T. C., Wu, Y. Y., Yeh, C. H., Chiang, W., Chuang, C. Y., & Kuo, H. C. The circular RNA circBIRC6 participates in the molecular circuitry controlling human pluripotency. Nature communications, 2017, 8(1), 1149.
40. Chen, S., Zong, Y., Hou, Z., Deng, Z., Xia, Z. Splicing factor ESRP1 derived circ_0068162 promotes the progression of oral squamous cell carcinoma via the miR-186/JAG axis. Carcinogenesis, 2024, 45(3):107-118.
41. Liu, D., Dredge, B. K., Bert, A. G., Pillman, K. A., Toubia, J., Guo, W., Dyakov, B. J. A., Migault, M. M., Conn, V. M., Conn, S. J., Gregory, P. A., Gingras, A. C., Patel, D., Wu, B., & Goodall, G. J. ESRP1 controls biogenesis and function of a large abundant multiexon circRNA. Nucleic acids research, 2024, 52(3), 1387–1403.
42. Chioccarelli, T., Falco, G., Cappetta, D., De Angelis, A., Roberto, L., Addeo, M., Ragusa, M., Barbagallo, D., Berrino, L., Purrello, M., Ambrosino, C., Cobellis, G., Pierantoni, R., Chianese, R., & Manfrevola, F. FUS driven circCNOT6L biogenesis in mouse and human spermatozoa supports zygote development. Cellular and molecular life sciences, 2021, 79(1), 50.
43. Chen, T., Wang, X., Li, C., Zhang, H., Liu, Y., Han, D., Li, Y., Li, Z., Luo, D., Zhang, N., Zheng, M., Chen, B., Wang, L., Zhao, W., & Yang, Q. CircHIF1A regulated by FUS accelerates triple-negative breast cancer progression by modulating NFIB expression and translocation. Oncogene, 2021, 40(15), 2756–2771.
44. Zheng, L., Tang, T., Wang, Z., Sun, C., Chen, X., Li, W., Wang, B. FUS-Mediated CircFGFR1 Accelerates the Development of Papillary Thyroid Carcinoma by Stabilizing FGFR1 Protein. Biochem Genet, 2024, 62(5), 3977–3995.
45. Yang, T., Shen, P., Chen, Q., Wu, P., Yuan, H., Ge, W., Meng, L., Huang, X., Fu, Y., Zhang, Y., Hu, W., Miao, Y., Lu, Z., & Jiang, K. FUS-induced circRHOBTB3 facilitates cell proliferation via miR-600/NACC1 mediated autophagy response in pancreatic ductal adenocarcinoma. Journal of experimental & clinical cancer research, 2021, 40(1), 261.
46. Ashwal-Fluss, R., Meyer, M., Pamudurti, N. R., Ivanov, A., Bartok, O., Hanan, M., Evantal, N., Memczak, S., Rajewsky, N., & Kadener, S. circRNA biogenesis competes with pre-mRNA splicing. Molecular cell, 2014, 56(1), 55–66.
47. Deng, F., Chen, C. B., Li, H., Huang, S., Xu, C., & Xiao, X. CircSlc17a5 controlled by VLDLR/QKI pathway regulated the choroidal angiogenesis. Biochimica et biophysica acta. Molecular cell research, 2024, 1871(7), 119802..
48. Gupta, S. K., Garg, A., Bär, C., Chatterjee, S., Foinquinos, A., Milting, H., Streckfuß-Bömeke, K., Fiedler, J., & Thum, T. Quaking Inhibits Doxorubicin-Mediated Cardiotoxicity Through Regulation of Cardiac Circular RNA Expression. Circulation research, 2018, 122(2), 246–254.
49. Conn, S. J., Pillman, K. A., Toubia, J., Conn, V. M., Salmanidis, M., Phillips, C. A., Roslan, S., Schreiber, A. W., Gregory, P. A., & Goodall, G. J. The RNA binding protein quaking regulates formation of circRNAs. Cell, 2015, 160(6), 1125–1134.
50. Huang, C., Yang, Y., Wang, X., Chen, S., Liu, Z., Li, Z., Tang, X., & Zhang, Q. PTBP1-mediated biogenesis of circATIC promotes progression and cisplatin resistance of bladder cancer. International journal of biological sciences, 2024, 20(9), 3570–3589.
51. Hu, H., Shen, S., Wu, J., Ma, L. CircTOP1 targeted regulation of PTBP1 expression promotes the progression of coronary artery calcification. Exp Cell Res, 2024, 440(2):114147.

52. Xu, P., Zhang, J., Wang, M., Liu, B., Li, R., Li, H., Zhai, N., Liu, W., Lv, C., & Song, X. hnRNPL-activated circANKRD42 back-splicing and circANKRD42-mediated crosstalk of mechanical stiffness and biochemical signal in lung fibrosis. Molecular therapy : the journal of the American Society of Gene Therapy, 2022, 30(6), 2370–2387.
53. Fei, T., Chen, Y., Xiao, T., Li, W., Cato, L., Zhang, P., Cotter, M. B., Bowden, M., Lis, R. T., Zhao, S. G., Wu, Q., Feng, F. Y., Loda, M., He, H. H., Liu, X. S., & Brown, M. Genome-wide CRISPR screen identifies HNRNPL as a prostate cancer dependency regulating RNA splicing. Proceedings of the National Academy of Sciences of the United States of America, 2017, 114(26), E5207–E5215.
54. Gao, P., Yang, Y., Li, X., Zhao, Q., Liu, Y., Dong, C., Zhang, Y., & Liu, D. Circular RNA hsa_circ_0098181 inhibits metastasis in hepatocellular carcinoma by activating the Hippo signaling pathway via interaction with eEF2. Annals of hepatology, 2023, 28(5), 101124.
55. Kong, Y., Luo, Y., Zheng, S., Yang, J., Zhang, D., Zhao, Y., Zheng, H., An, M., Lin, Y., Ai, L., Diao, X., Lin, Q., Chen, C., & Chen, R. Mutant KRAS Mediates circARFGEF2 Biogenesis to Promote Lymphatic Metastasis of Pancreatic Ductal Adenocarcinoma. Cancer research, 2023, 83(18), 3077–3094.
56. Shen, P., Yang, T., Chen, Q., Yuan, H., Wu, P., Cai, B., Meng, L., Huang, X., Liu, J., Zhang, Y., Hu, W., Miao, Y., Lu, Z., & Jiang, K. CircNEIL3 regulatory loop promotes pancreatic ductal adenocarcinoma progression via miRNA sponging and A-to-I RNA-editing. Molecular cancer, 2021, 20(1), 51.
57. Shi, L., Yan, P., Liang, Y., Sun, Y., Shen, J., Zhou, S., Lin, H., Liang, X., & Cai, X. Circular RNA expression is suppressed by androgen receptor (AR)-regulated adenosine deaminase that acts on RNA (ADAR1) in human hepatocellular carcinoma. Cell death & disease, 2017, 8(11), e3171.
58. Zhang, Y., Li, X., Zhang, J., Mao, L., Wen, Z., Cao, M., Mu, X. USF1 regulated circPRDM4 modulates tumorigenesis and immune escape in chemoresistant cervical cancer. J Cell Mol Med, 2024, 28(5):e17945.
59. Ai, J., Zhang, W., Deng, W., Yan, L., Zhang, L., Huang, Z., Wu, Z., Ai, J., & Jiang, H. A hsa_circ_001726 axis regulated by E2F6 contributes to metastasis of hepatocellular carcinoma. BMC cancer, 2024, 24(1), 14.
60. Jin, R., Li, H., Nan, S., Wang, H. FOXA1 co-activates circODC1 and ODC1 in HPV-positive cervical cancer cell growth. Syst Biol Reprod Med, 2024, 70(1):113-123.
61. Qin, C., Liu, S., Chen, W., Xue, D., Guo, T., Wu, B. HuR-induced circ_0082319 contributes to hepatocellular carcinoma by elevating PTK2 through miR-505-3p. Naunyn Schmiedebergs Arch Pharmacol, 2024, 397(5):3111-3126.
62. Ji, Y., Ni, C., Shen, Y., Xu, Z., Tang, L., Yu, F., Zhu, L., Lu, H., Zhang, C., Yang, S., & Wang, X. ESRP1-mediated biogenesis of circPTPN12 inhibits hepatocellular carcinoma progression by PDLIM2/ NF-κB pathway. Molecular cancer, 2024, 23(1), 143.
63. Han, Y., Meng, J., Ling, X., Pan, Z., Zhang, H., Zhong, B., Chen, S., Pang, J., Ma, Y., Chen, J., & Liu, L. DNMT1 regulates hypermethylation and silences hsa_circ_401351 in hydroquinone-induced malignant TK6 cells. Environmental toxicology, 2024 39(4), 2092–2101.
64. Lv, K., Xie, P., Yang, Q., Luo, M., Li, C. hsa_circ_0101050 regulated by ZC3H13 enhances tumorigenesis in papillary thyroid cancer via m6A modification. Heliyon, 2024, 10(12):e32913.
65. Feng, N., Ye, Y., Pan, Y., Kuang, B., Du, Y., Geng, N., Chen, C., Liu, K., Liang, L., Xian, M., Yang, Y., Li, X., Deng, L., Zhang, F., Kuang, L., Fan, M., Xie, Y., & Guo, F. The circUbqln1, regulated by XBP1s, interplays with 14-3-3ζ to inhibit collagen synthesis and promote osteoarthritis by controlling PRODH activity and proline metabolism. Journal of advanced research, 2024, 2090-1232(24)00007-9.
66. Chang, S., Wang, Y., Wang, X., Liu, H., Zhang, T., Zheng, Y., Wang, X., Shan, G., & Chen, L. HNRNPD regulates the biogenesis of circRNAs and the ratio of mRNAs to circRNAs for a set of genes. RNA biology, 2024, 21(1), 1–15.
67. Conn, V. M., Gabryelska, M., Marri, S., Stringer, B. W., Ormsby, R. J., Penn, T., Poonnoose, S., Kichenadasse, G., & Conn, S. J. SRRM4 Expands the Repertoire of Circular RNAs by Regulating Microexon Inclusion. Cells, 2020, 9(11), 2488.

68. Aktaş, T., Avşar Ilık, İ., Maticzka, D., Bhardwaj, V., Pessoa Rodrigues, C., Mittler, G., Manke, T., Backofen, R., & Akhtar, A. DHX9 suppresses RNA processing defects originating from the Alu invasion of the human genome. Nature, 2017, 544(7648), 115–119.
69. Knupp, D., Cooper, D.A., Saito, Y., Darnell, R.B., Miura, P. NOVA2 regulates neural circRNA biogenesis. Nucleic Acids Res, 2021, 49(12):6849-6862.
70. Xie, X., Sun, F. K., Huang, X., Wang, C. H., Dai, J., Zhao, J. P., Fang, C., & He, W. A circular RNA, circSMARCA5, inhibits prostate cancer proliferative, migrative, and invasive capabilities via the miR-181b-5p/miR-17-3p-TIMP3 axis. Aging, 2021, 13(15), 19908–19919.
71. Dai, X., Zhang, N., Cheng, Y., Yang, T., Chen, Y., Liu, Z., Wang, Z., Yang, C., & Jiang, Y. RNA-binding protein trinucleotide repeat-containing 6A regulates the formation of circular RNA circ0006916, with important functions in lung cancer cells. Carcinogenesis, 2018, 39(8), 981–992.
72. Khan, M. A., Reckman, Y. J., Aufiero, S., van den Hoogenhof, M. M., van der Made, I., Beqqali, A., Koolbergen, D. R., Rasmussen, T. B., van der Velden, J., Creemers, E. E., & Pinto, Y. M. RBM20 Regulates Circular RNA Production From the Titin Gene. Circulation research, 2016, 119(9), 996–1003.
73. Dong, W., Dai, Z. H., Liu, F. C., Guo, X. G., Ge, C. M., Ding, J., Liu, H., & Yang, F. The RNA-binding protein RBM3 promotes cell proliferation in hepatocellular carcinoma by regulating circular RNA SCD-circRNA 2 production. EBioMedicine, 2019, 45, 155–167.
74. Li, T.R., Jia, Y.J., Wang, Q., Shao, X.Q., Lv, R.J. Circular RNA: a new star in neurological diseases. Int J Neurosci, 2017, 127(8):726-734.
75. Zeng, K., Chen, X., Xu, M., Liu, X., Hu, X., Xu, T., Sun, H., Pan, Y., He, B., & Wang, S. Retraction Note: CircHIPK3 promotes colorectal cancer growth and metastasis by sponging miR-7. Cell death & disease, 2024, 15(5), 372.
76. Zhang, Y., Zhang, X. O., Chen, T., Xiang, J. F., Yin, Q. F., Xing, Y. H., Zhu, S., Yang, L., & Chen, L. L. Circular intronic long noncoding RNAs. Molecular cell, 2013, 51(6), 792–806.
77. Liu, C. X., Li, X., Nan, F., Jiang, S., Gao, X., Guo, S. K., Xue, W., Cui, Y., Dong, K., Ding, H., Qu, B., Zhou, Z., Shen, N., Yang, L., & Chen, L. L. Structure and Degradation of Circular RNAs Regulate PKR Activation in Innate Immunity. Cell, 2019, 177(4), 865–880.e21.
78. Schaeffer, D., Tsanova, B., Barbas, A., Reis, F. P., Dastidar, E. G., Sanchez-Rotunno, M., Arraiano, C. M., & van Hoof, A. The exosome contains domains with specific endoribonuclease, exoribonuclease and cytoplasmic mRNA decay activities. Nature structural & molecular biology, 2009, 16(1), 56–62.
79. Jia, R., Xiao, M. S., Li, Z., Shan, G., & Huang, C. Defining an evolutionarily conserved role of GW182 in circular RNA degradation. Cell discovery, 2019, 5, 45.
80. Hansen, T. B., Wiklund, E. D., Bramsen, J. B., Villadsen, S. B., Statham, A. L., Clark, S. J., & Kjems, J. miRNA-dependent gene silencing involving Ago2-mediated cleavage of a circular antisense RNA. The EMBO journal, 2011, 30(21), 4414–4422.
81. Stagsted, L. V. W., O'Leary, E. T., Ebbesen, K. K., & Hansen, T. B. The RNA-binding protein SFPQ preserves long-intron splicing and regulates circRNA biogenesis in mammals. eLife, 2021, 10, e63088.
